# Marilones A–C, phthalides from the sponge-derived fungus *Stachylidium* sp.

**DOI:** 10.3762/bjoc.7.192

**Published:** 2011-12-05

**Authors:** Celso Almeida, Stefan Kehraus, Miguel Prudêncio, Gabriele M König

**Affiliations:** 1 Institute for Pharmaceutical Biology, University of Bonn, Nussallee 6, D-53115 Bonn, Germany; 2 Instituto de Medicina Molecular, Malaria Unit, Faculdade de Medicina, Universidade de Lisboa, Av. Prof. Egas Moniz, 1649-028 Lisboa, Portugal

**Keywords:** marine fungi, natural products, phthalides, polyketides

## Abstract

The marine-derived fungus *Stachylidium* sp. was isolated from the sponge *Callyspongia* sp. cf. *C. flammea.* Culture on a biomalt medium supplemented with sea salt led to the isolation of three new phthalide derivatives, i.e., marilones A–C (**1**–**3**), and the known compound silvaticol (**4**). The skeleton of marilones A and B is most unusual, and its biosynthesis is suggested to require unique biochemical reactions considering fungal secondary metabolism. Marilone A (**1**) was found to have antiplasmodial activity against *Plasmodium berghei* liver stages with an IC_50_ of 12.1 µM. Marilone B (**2**) showed selective antagonistic activity towards the serotonin receptor 5-HT_2B_ with a *K*_i_ value of 7.7 µM.

## Introduction

Phthalides are a class of structurally very diverse secondary metabolites with more than 180 naturally occurring compounds described [1]. They are produced by a wide range of organisms, i.e., by marine and terrestrial fungi belonging to genera such as *Ascochyta* [2], *Aspergillus* [3–5], *Alternaria* [6], *Penicillium* [7], *Hericium* [8] or *Talaromyces* [9], but also by plants and liverworts [1].

Phthalides exhibit an equally broad spectrum of bioactivity, including modulation of the central nervous system, protection against brain eschemia, modulation of platelet aggregation and cardiac function, inhibition of smooth muscle cell proliferation, anti-angina activity, and smooth muscle relaxation, as well as antibacterial, antifungal, antiviral and phytotoxic activity [1]. The medically most important member of this family of natural products is mycophenolic acid, initially isolated from *Penicillium brevicompactum*, and used in the form of its derivative mycophenolate mofetil as an immunosuppressant drug [10].

**Scheme 1:**
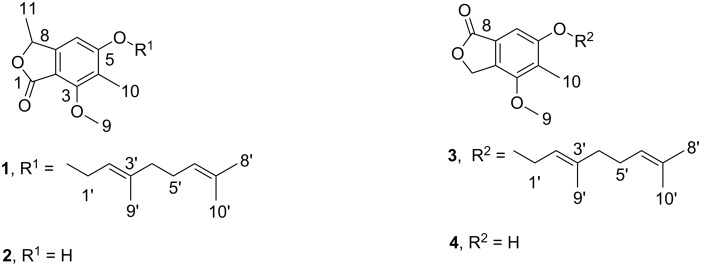
Secondary metabolites **1**–**4** isolated from *Stachylidium* sp.

During our search for new natural products produced from the marine-derived fungus *Stachylidium* sp., several phthalide derivatives, i.e., marilones A–C, were isolated from a culture on agar-BMS media supplemented with artificial sea salt (Scheme 1). Albeit phthalide-like structures are not rare, the structural skeleton of marilones A and B is most unusual, and its biosynthesis is suggested to require unique reactions in fungal secondary metabolism. Marilone A (**1**) exhibited antiplasmodial activity against *Plasmodium berghei* with an IC_50_ of 12.1 µM. Marilone B (**2**) showed a specific antagonistic effect on the serotonin receptor 5-HT_2B_ with a *K*_i_ value of 7.7 µM.

## Results and Discussion

The molecular formula of **1** was deduced by accurate mass measurement (HRMS–EI) to be C_21_H_28_O_4_, requiring eight degrees of unsaturation. The ^13^C NMR and DEPT135 spectra contained 21 carbon resonances, including six resulting from methyl groups, three from sp^2^ methines, and one from an sp^3^ methine, whereas a further three signals resulted from methylene groups, and eight resonances were assigned to quaternary carbons (Table 1, Table 2 and Supporting Information File 1). The ^1^H NMR spectrum of **1** displayed a singlet resonance for the aromatic methine (6-CH) at δ 6.95 indicating, together with UV and ^13^C NMR data, the presence of a penta-substituted benzene ring. The methyl group 10-CH_3_ (δ_C_ 8.8) was linked to C-4 of the aromatic ring due to heteronuclear long range correlations of the methyl protons with C-3, C-4 and C-5. The methoxy group 9-OCH_3_ (δ_H_ 3.98) had a heteronuclear long range correlation to C-3 of the aromatic ring, thus, clearly delineating its position. Besides the aromatic proton 6-H, the ^1^H NMR spectrum showed two further resonance signals in the downfield shifted region (δ_H_ 5.52 and 5.09) indicating, together with ^13^C NMR and ^1^H/^13^C HMBC data, the presence of a geranyl substituent. The C-1’ to C-10’ part of the molecule was deduced from two proton coupling spin systems observed in the ^1^H/^1^H COSY spectrum, namely 1’-H_2_ to 2’-H (*J* = 6.6 Hz) and 4’-H_2_ to 6’-H through 5’-H_2_. ^1^H/^13^C HMBC data showed correlations from 9’-H_3_ to C-2’, C-3’ and C-4’, and from methyl protons 8’-H_3_ and 10’-H_3_ to C-6’and C-7’, disclosing a geranyl fragment. Based on literature comparisons we established the configuration of Δ^2’/3’^ as *E* [11]. The aromatic quaternary carbon C-5 (δ_C_ 164.2) had a carbon resonance that indicated a connection to an oxygen atom. The monoterpenyl substituent was established to be connected to C-5 through an oxygen atom, based on the heteronuclear long range correlations of 1’-H_2_ (δ_H_ 4.69, 4.74) to C-5.

**Table 1:** ^13^C NMR spectroscopic data for compounds **1**, **2**, and **3**.

	**1**	**2**	**3**

pos.	δ_C_, mult.^a, b^	δ_C_, mult.^a, b^	δ_C_, mult.^a, b^

1	168.2, qC	168.2, qC	68.9, CH_2_
2	110.0, qC	109.1, qC	128.4, qC
3	158.0, qC	158.9, qC	153.9, qC
4	120.4, qC	118.8, qC	124.8, qC
5	164.2, qC	163.2, qC	159.7, qC
6	100.8, CH	103.6, CH	102.0, CH
7	154.2, qC	153.9, qC	125.8, qC
8	77.0, CH	76.6, CH	171.1, qC
9	62.1, CH_3_	62.0, CH_3_	59.3, CH_3_
10	8.8, CH_3_	8.6, CH_3_	9.8, CH_3_
11	20.9, CH_3_	21.0, CH_3_	–
1'	66.5, CH_2_	–	66.4, CH_2_
2'	120.1, CH	–	120.5, CH
3'	142.1, qC	–	141.6, qC
4'	40.1, CH_2_	–	40.1, CH_2_
5'	26.9, CH_2_	–	26.9, CH_2_
6'	124.6, CH	–	124.6, CH
7'	132.1, qC	–	132.1, qC
8'	25.8, CH_3_	–	25.8, CH_3_
9'	16.7, CH_3_	–	16.7, CH_3_
10'	17.7, CH_3_	–	17.7, CH_3_

^a^Acetone-*d*_6_, 75.5 MHz. ^b^Implied multiplicities determined by DEPT.

**Table 2:** ^1^H NMR spectroscopic data for compounds **1**, **2**, and **3**.

	**1**	**2**	**3**

pos.	δ_H_^a, b^ (*J* in Hz)	δ_H_^a, b^ (*J* in Hz)	δ_H_^a, b^ (*J* in Hz)

1	–	–	5.50, s
2	–	–	–
3	–	–	–
4	–	–	–
5	–	–	–
6	6.95, s	6.75, s	7.03, s
7	–	–	–
8	5.43, q (6.6)	5.37, q (6.6)	–
9	3.98, s	3.98, s	3.96, s
10	2.09, s	2.10, s	2.15, s
11	1.54, d (6.6)	1.48, d (6.6)	–
1’	a: 4.69, dd (6.6, 12.1) b: 4.74, dd (6.6, 12.1)	–	4.71, d (6.6)
2’	5.52, t (6.6)	–	5.51, t (6.6)
3'	–	–	–
4'	2.10, m	–	2.10, m
5'	2.13, m	–	2.14, m
6'	5.09, m	–	5.10, m
7'	–	–	–
8'	1.63, br s	–	1.63, br s
9'	1.76, br s	–	1.78, br s
10'	1.58, br s	–	1.58, br s

^a^Acetone-*d*_6_, 300 MHz. ^b^Assignments are based on extensive 1D and 2D NMR experiments (HMBC, HSQC, COSY).

The ^1^H/^13^C HMBC spectrum exhibited a correlation from 6-H to C-8. Furthermore, the ^13^C NMR resonance of C-8 at δ 77.0 was found to be characteristic for a carbon bound to an oxygen atom. The ^1^H/^1^H COSY spectrum showed a coupling of 8-H with 11-H_3_ (*J* = 6.6 Hz), and the ^1^H/^13^C HMBC spectrum contained correlations from 8-H to C-6, C-7 and C-2 of the penta-substituted aromatic ring, as well as to the carbonyl carbon C-1. Ring double bond equivalents required a second ring within compound **1**, and together with heteronuclear correlations of 8-H to C-1 and the carbon resonance of C-1 at δ_C_ 168.2 indicating the presence of a carbonyl group, this gave evidence for a C-8-methylated phthalide skeleton, i.e., the C-1 to C-11 part of the structure. Since the resonance signal for 6-H did not show heteronuclear long range correlations to that of the carbonyl C-1, but instead correlated with the sp^3^ methine C-8, the carbonyl group was assigned at C-1. In this way, a phthalide-nucleus identical to that of the known natural product nidulol was formed [6]. To further prove that the carbonyl group is positioned at C-1 and not at C-8 of **1**, ^1^H NMR spectra of **1** were compared with those of nidulol and silvaticol (**4**) (and derivatives, see Supporting Information File 1). The latter are known regioisomeric phthalides with the carbonyl group at C-1 and C-8, respectively. Differences in ^1^H NMR resonances can be discerned especially for 6-H, resonating at δ_H_ 6.59 (CDCl_3_) for nidulol and δ_H_ 7.04 (CDCl_3_) for silvaticol [6]. The ^1^H NMR spectrum of **1** (δ_H_ 6.54 in CDCl_3_) was shown to be similar to that of nidulol and the nidulol derivative 5-(3',3'-dimethylallyloxy)-7-methoxy-6-methylphthalide with 6-H resonating at δ_H_ 6.62 (see Supporting Information File 1) [5]. For compound **1** the trivial name marilone A is suggested.

The molecular formula of **2** was deduced by accurate mass measurement (HRMS–EI) to be C_11_H_12_O_4_, requiring six sites of unsaturation. The NMR spectral data (see Table 1, Table 2 and Supporting Information File 1) indicated that compound **2** is identical to **1**, except for the missing geranyl moiety attached to the hydroxy group at C-5. We propose the trivial name marilone B for compound **2**.

The molecular formula of **3**, was deduced by accurate mass measurement (HRMS–EI) to be C_20_H_26_O_4_, requiring eight degrees of unsaturation. The spectroscopic data of **3** revealed that the compound is also very similar to **1** (Table 1, Table 2 and Supporting Information File 1). In contrast to compound **1**, however, resonance signals for a methylene group, i.e., 1-CH_2_ (δ_C_ 68.9) were found in the NMR spectra, instead of those for a methine (8-CH) and methyl group (11-CH_3_) as in **1**. The resonance signal for 6-H did not have a heteronuclear long range correlation to C-1, but correlated with the carbonyl carbon C-8. Hence, the location of carbonyl group in **3** was assigned to C-8, thus, forming a phthalide-nucleus as present in silvaticol (**4**). For compound **3** the name marilone C is suggested.

Spectroscopic data of **4** were determined to be identical to those of silvaticol (Supporting Information File 1) [6].

Compounds **1** and **2** possess a single chiral center at C-8. The measurement of the specific optical rotation for these compounds yielded values close to zero, and furthermore, the CD measurements showed hardly any CD effect for the referred compounds. This was expected at around 260 nm due to the proximity of the chiral center to the chromophoric penta-substituted benzene ring. We thus assumed the presence of racemic mixtures for these chiral compounds. Extensive trials to separate the enantiomers, employing three different HPLC chiral stationary phases, were unsuccessful. However, the presence of racemic mixtures was proven for the analogous, nitrogen-containing compounds, i.e., phthalimidine derivatives isolated from the same fungus (Almeida et al*.*, unpublished data).

Marilones A, B and C (**1**–**3**) were tested for antiplasmodial activity*,* and marilone A exhibited an IC_50_ of 12.1 µM against the liver stage of *Plasmodium berghei* (see Supporting Information File 1). Interestingly, marilone C (**3**) showed no activity at 25 µM concentration, indicating that the methyl group 11-CH_3_ and/or the position of the ketone functionality is essential for this bioactivity.

Marilones A, B, and C (**1**–**3**) were also tested for cytotoxic activity towards three cancer cell lines (NCI-H460, MCF7 and SF268). Marilone A and C (**1**, **3**) showed weak antiproliferative activity with an average GI_50_ of 36.7 and 26.6 µM, respectively (see Supporting Information File 1).

Marilone B (**2**) was assayed in a panel of 44 psychoactive receptors, including 11 serotonin receptors, and marilone B showed a specific antagonistic effect on the serotonin receptor 5-HT_2B_ with a *K*_i_ value of 7.7 µM.

Compounds **1**–**4** were further evaluated for antiviral activity, for inhibition of protein kinases and proteases, for growth inhibition of antibiotic-resistant *Mycobacterium tuberculosis* as well as further microbial pathogens, for activity in an antidiabetic activity assay panel, in a 3T3-L1 murine adipocyte assay, and in a NF-κB protein complex assay, but they exhibited no activity (see detailed description in Supporting Information File 1).

Phthalide derivatives are compounds of the polyketide metabolism, which are common in nature [1]. Secondary metabolites **1** and **2** discovered in the marine-derived *Stachylidium* sp. were found to be derivatives of the natural product nidulol, whilst compound **3** was a derivative of silvaticol (**4**) (see Supporting Information File 1), formerly described from the fungus *Aspergillus silvaticus* [6]. Nidulol and silvaticol (**4**) are regioisomeric compounds and differ in terms of the position of the carbonyl group, which is either placed *peri* to the aromatic hydrogen, as in **3** and **4**, or it is positioned *peri* to the aromatic methoxy moiety, e.g., in **1** and **2**. The *Stachylidium* species investigated here is thus able to produce both types of phthalides, which are suspected to differ significantly in terms of their biosynthesis (Supporting Information File 1; Figure S15).

Whereas compound **3** is simply the O-prenylated form of silvaticol (**4**), the nidulol derivatives **1** and **2** are distinguished by an additional methyl substituent (11-CH_3_) at C-8. In terms of biosynthesis, i.e., polyketide metabolism, this substitution is most unusual for phthalides and, to the best of our knowledge, it was only found once in dimethoxydimethylphthalide (DDP) [12].

Biosynthetic studies focusing on phthalide structures, e.g., for mycophenolic acid [13], nidulol and silvaticol [9], were previously performed by means of feeding experiments with labeled precursors, evidencing the tetraketide nature of the phthalide nucleus (Supporting Information File 1; Figure S15). Compounds **1**–**4**, possess a basic skeleton which is related to that of the well-known tetraketide 3-methyl-orsellinic acid [14]. Closing of the lactone ring would for compounds **1** and **2** require the oxidation of C-8 to obtain a hydroxy group, which could subsequently form a lactone with the C-1 carboxy group (Supporting Information File 1; Figure S15A). In contrast to that for **3** and **4**, a reduction of the C-1 carboxy group to an alcoholic function and an oxidation of C-8 to a carboxylic function would be required (Supporting Information File 1; Figure S15 B).

Most intriguing, however, is that in compounds **1** and **2** the acetate-derived methyl group 8-CH_3_ in the methyl-orsellinic acid precursor would be replaced by an ethyl group. Thus, the biosynthesis seems to require either a propionate starter unit (see C in Figure S15; Supporting Information File 1) or a methylation (e.g., via a SAM-dependent methyl-transferase) at C-8 (see D in Figure S15; Supporting Information File 1). A third possibility would be the loss of a carbon atom from a pentaketide intermediate. To our knowledge, to date propionate as a starter unit was only described for pseurotin A and austrocorticinic acid in fungal polyketide metabolism [15–16]. Feeding experiments are under way in order to determine the building blocks for these molecules.

It is worthwhile to mention that marilones were produced solely on solid biomalt medium (BMS) supplemented with sea salt, whereas in other media such as Czapek or YPM no phthalides were formed.

## Experimental

**General experimental procedures.** Optical rotations were measured on a Jasco DIP 140 polarimeter. UV and IR spectra were obtained with a Perkin-Elmer Spectrum BX instrument. All NMR spectra were recorded in MeOD or (CD_3_)_2_CO on a Bruker Avance 300 DPX spectrometer. Spectra were referenced to residual solvent signals with resonances at δ_H/C_ 3.35/49.0 for MeOD and δ_H/C_ 2.04/29.8 for (CD_3_)_2_CO. HRMS–EI were recorded on a Finnigan MAT 95 spectrometer. HRMS–ESI were recorded on a Bruker Daltonik micrOTOF-Q time-of-flight mass spectrometer with ESI source. HPLC was carried out on a system composed of a Waters 515 pump together with a Knauer K-2300 differential refractometer. HPLC columns were from Knauer (250 × 8 mm, Eurospher-100 Si and 250 × 8 mm, Eurospher-100, C18, 5 μm; flow 2 mL/min) and Macherey-Nagel (Nucleodur C18 EC Isis 250 × 4.6 mm, 5 μm, flow: 1 mL/min). Merck silica gel 60 (0.040–0.063 mm, 70–230 mesh) was used for vacuum liquid chromatography (VLC). Columns were wet-packed under vacuum with petroleum ether (PE). Before applying the sample solution, the columns were equilibrated with the first designated eluent. Standard columns for crude extract fractionation had dimensions of 13 × 4 cm.

**Fungal material**. The marine-derived fungus *Stachylidium* sp*.* was isolated from the sponge *Callyspongia* sp. cf. *C. flammea* (collected at Bear Island, Sydney, Australia) and identified by P. Massart and C. Decock, BCCM/MUCL, Catholic University of Louvain, Belgium. A specimen is deposited at the Institute for Pharmaceutical Biology, University of Bonn, isolation number “293K04”, culture collection number “220”.

**Cultivation, extraction and isolation.** Compounds **1**–**4** were isolated from a 60 days culture (12 L) of *Stachylidium* sp. on an agar–biomalt medium supplemented with sea salt (BMS). An extraction with 5 L EtOAc yielded 5.9 g of extract, which was subjected to a VLC fractionation in an open column with silica as solid phase and a gradient solvent system with petroleum ether/acetone of 10:1, 5:1, 2:1, 1:1, 100% acetone and 100% MeOH, resulting in six VLC fractions. Compounds **1** and **3** were isolated from VLC fraction 1. VLC fraction 1 was again fractionated using petroleum ether/acetone 90:1 and 10:1 in order to eliminate fatty acid content of the sample. The VLC fraction 10:1 was subjected to NP-HPLC fractionation using petroleum ether/acetone 30:1 to yield a mixture of both compounds (subfraction 4 of 7). Further fractionation using MeOH/H_2_O 8:2 (RP-HPLC, Isis column) yielded compound **1** (subfraction 1 of 2; 19 mg, *t*_R_ 13 min) and compound **3** (subfraction 2 of 2; 14.2 mg, *t*_R_ 15 min).

Compounds **2** and **4** were isolated from VLC fraction 2, followed by NP-HPLC fractionation using PE/acetone 11:1 to yield a mixture of both compounds (fraction 6 of 7). Further fractionation using MeOH/H_2_O 4:6 (RP-HPLC, Isis column) yielded compound **2** (fraction 1 of 2; 5.2 mg, *t*_R_ 35 min) and the known compound **4**, silvaticol (fraction 2 of 2; 5.6 mg, *t*_R_ 38 min).

**Marilone A (1)**: transparent oil (1.6 mg/L, 0.32%); UV (MeOH) λ_max_, nm (log ε): 219 (4.38), 261 (2.95); IR (ATR) ν_max_: 2964, 1745, 1603 cm^−1^; ^1^H NMR and ^13^C NMR (Table 1 and Table 2); LRMS–EI (*m/z*): 344.2 [M]^+^; HRMS–EI (*m/z*): [M]^+^ calcd for C_21_H_28_O_4_, 344.1988; found, 344.1996.

**Marilone B (2)**: white amorphous solid (0.4 mg/L, 0.09%); UV (MeOH) λ_max_, nm (log ε): 215 (4.09), 260 (2.83); IR (ATR) ν_max_: 3238 (br), 2931, 1708, 1601 cm^−1^; ^1^H NMR and ^13^C NMR (Table 1 and Table 2); LRMS–EI (*m/z*): 208.1 [M]^+^; HRMS–EI (*m/z*): [M]^+^calcd for C_11_H_12_O_4_, 208.0736; found, 208.0737.

**Marilone C (3)**: transparent oil (1.3 mg/L, 0.27%); UV (MeOH) λ_max_, nm (log ε): 223 (4.10), 255 (2.80); IR (ATR) ν_max_: 2921, 1760, 1619 cm^−1^; ^1^H NMR and ^13^C NMR (Table 1 and Table 2); LRMS–EI (*m/z*): 330.2 (M)^+^; HRMS–EI (*m/z*): [M]^+^ calcd for C_20_H_26_O_4_, 330.1831; found, 330.1833.

### Methodology for the performed biological assays

The referred compounds were tested in antibacterial (*Escherichia coli*, *Bacillus megaterium*), antifungal (*Mycotypha microspora*, *Eurotium rubrum*, and *Microbotryum violaceum*), and antialgal (*Chlorella fusca*) assays as described before [17–18]. The inhibition of the following panel of proteases inhibition assays (chymotrypsin, trypsin, the protease elastase HLE, papain, porcine cease and acetylcholine esterase) were performed according to Neumann et al. [19]. Compounds were tested for protein kinase inhibition assays (DYRK1A and CDK5) according to Bettayeb et al. [20]. The triglyceride accumulation inhibition in the 3T3-L1 murine adipocytes assay was performed as described by Shimokawa et al. [21]. Cytotoxic activity assay against a panel of three cancer cell lines, NCI-H460, MCF7 and SF268 at the 100 µM level was performed according to Saroglou et al. [22] and Monks et al. [23]. Compounds were tested for antiplasmodial activity against *Plasmodium berghei* liver stages as described by Ploemen et al. [24]. Inhibition of the viral HIV-1- and HIV-2-induced cytopathogenic effect in MT-4 cells assays was performed according to Pannecouque et al. [25] and Zhan et al. [26]. Severe Acute Respiratory Syndrome coronavirus (SARS) assays were performed according to Kumaki et al. [27], the Herpes Simplex Virus-2 (HSV-2) activity assays according to Harden et al. [28], the Respiratory Syncytial virus (RSV) activity assays according to Barnard et al. [29–30], the Influenza viruses A and B (Flu A and B) activity assays as described by Sidwell and Smee [31], and the Hepatitis B virus was performed according to Sells et al. [32] and Korba and Gerin [33]. The activity assays against two strains of antibiotic resistant *Mycobacterium tuberculosis* were performed according to Bauer et al. [34]. The methodology for the inhibition of the NF-κB protein complex is described by Schumacher et al. [35]. The compounds were tested against a panel of antidiabetic activity assays as described by Marrapodi and Chiang [36], Dey et al. [37] and Seale et al. [38]. The binding assays against a panel of 44 psychoactive receptors (activity considered with at least 50% inhibition at the 10 μM level against 5-HT_1A_, 5-HT_1B_, 5-HT_1D_, 5-HT_1E_, 5-HT_2A_, 5-HT_2B_, 5-HT_2C_, 5-HT_3_, 5-HT_5A_, 5-HT_6_, 5-HT_7_, α_1A_, α_1B_, α_1D_, α_2A_, α_2B_, α_2C_, β_1_, β_2_, β_3_, BZP Rat Brain Site, D_1_, D_2_, D_3_, D_4_, D_5_, DAT, δ, κ, μ, GABA_A_, H_1_, H_2_, H_3_, H_4_, M_1_, M_2_, M_3_, M_4_, M_5_, NET, SERT, σ_1_, σ_2_) are fully described [39].

## Supporting Information

File 1 Spectroscopic data and other relevant information for compounds **1**–**4**.
