# Supplementary material for: Marilones A–C, phthalides from the sponge-derived fungus Stachylidium sp
Source: Beilstein J Org Chem. 2011 Dec 5;7:1636–42. doi: 10.3762/bjoc.7.192 (PMC3252867; doi:10.3762/bjoc.7.192)

Supporting Information

for

Marilones A–C, phthalides from the sponge-derived fungus *Stachylidium* sp.

Celso Almeida[1], Stefan Kehraus[1], Miguel Prudêncio[2] and Gabriele M. König*[1]

Address: [1]Institute for Pharmaceutical Biology, University of Bonn, Nussallee 6, D-53115 Bonn, Germany and [2] Instituto de Medicina Molecular, Malaria Unit, Faculdade de Medicina, Universidade de Lisboa, Av. Prof. Egas Moniz, 1649-028 Lisboa, Portugal

Email: Gabriele M. König* - g.koenig@uni-bonn.de

*Corresponding author

Spectroscopic data and other relevant information for compounds 1–4.

Figure S1 ^1H NMR (300 MHz, CD_3COCD_3) of compound **1**…………………………3
Figure S2 ^{13}C NMR (75 MHz, CD_3COCD_3) of compound **1**………………………….3
Figure S3 ^1H NMR (300 MHz, CD_3COCD_3) of compound **2**…………………………4
Figure S4 ^{13}C NMR (75 MHz, CD_3COCD_3) of compound **2**………………………….4
Figure S5 ^1H NMR (300 MHz, CD_3COCD_3) of compound **3**…………………………5
Figure S6 ^{13}C NMR (75 MHz, CD_3COCD_3) of compound **3**………………………….5
Figure S7 Key HMBC correlations for compounds **1** – **3**……………………………..6
Figure S8 HREIMS data for compound **1**..6
Figure S9 HREIMS data for compound **2**……………………………………………..7
Figure S10 HREIMS data for compound **3**……………………………………………..7
Figure S11 Antiproliferative activity data for compounds **1** and **3**……………………..8
Figure S12a–c Antiplasmodial activity data for compounds **1** and **3**……………………...9
Figure S13 Ki value determination of the antagonistic effect of Marilone B against the serotonin receptor 5HT2B…………………………………………………11
S14 Detailed description of the negative biological activity assays………….....11
Table S1 1D and 2D NMR spectroscopic data for compound **1**……………………..13
Table S2 1D and 2D NMR spectroscopic data for compound **2**……………………..14
Table S3 1D and 2D NMR spectroscopic data for compound **3**……………………..15
Table S4 ^1H-NMR spectroscopic data comparison of compound **4** with silvaticol and nidulol………………………………………………………………………16
Table S5 ^1H-NMR spectroscopic data comparison of compounds **1** and **3** with silvaticol and nidulol derivatives……………………………………………17
Figure S15 Tetraketide nature of the phthalides nidulol (A) and silvaticol (B), and hypothetical biosynthetic pathways (C, D) for marilones A and B (**1**, **2**)…..18

Figure S1: ^{1}H NMR (300 MHz, CD_3COCD_3) of compound **1**.

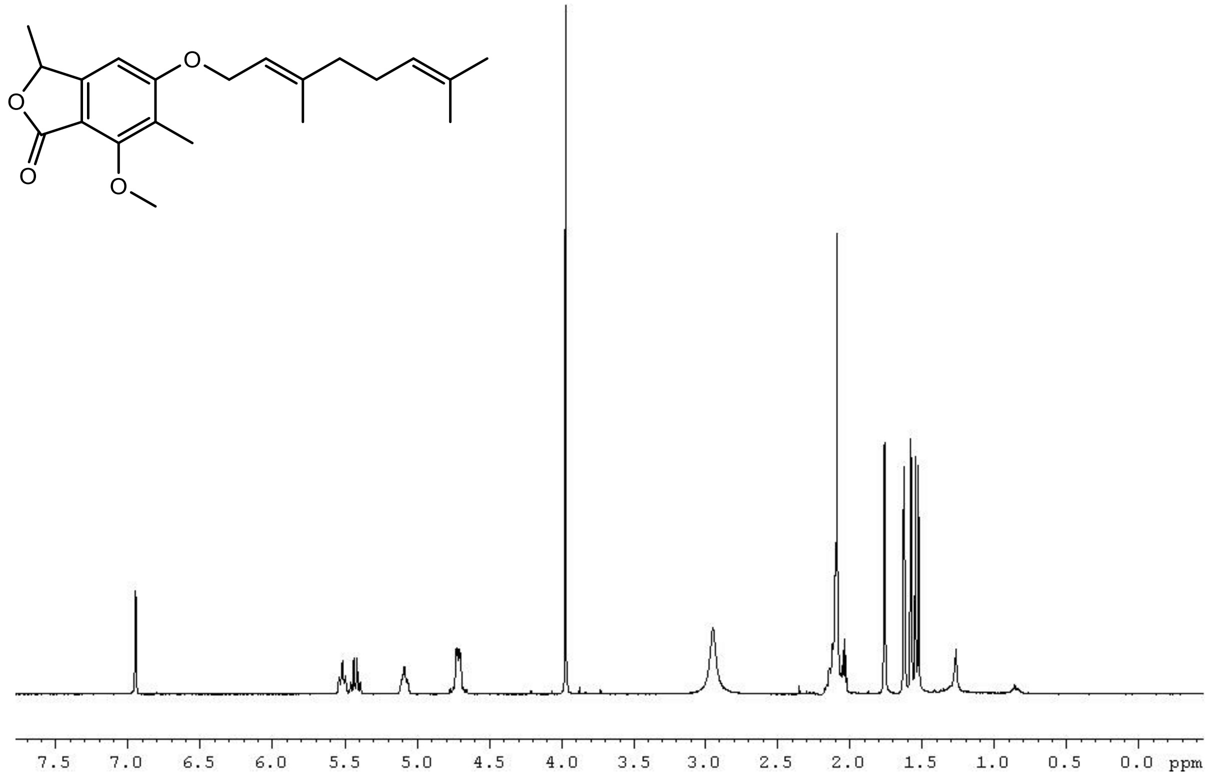

Figure S2: ^{13}C NMR (75 MHz, CD_3COCD_3) of compound **1**.

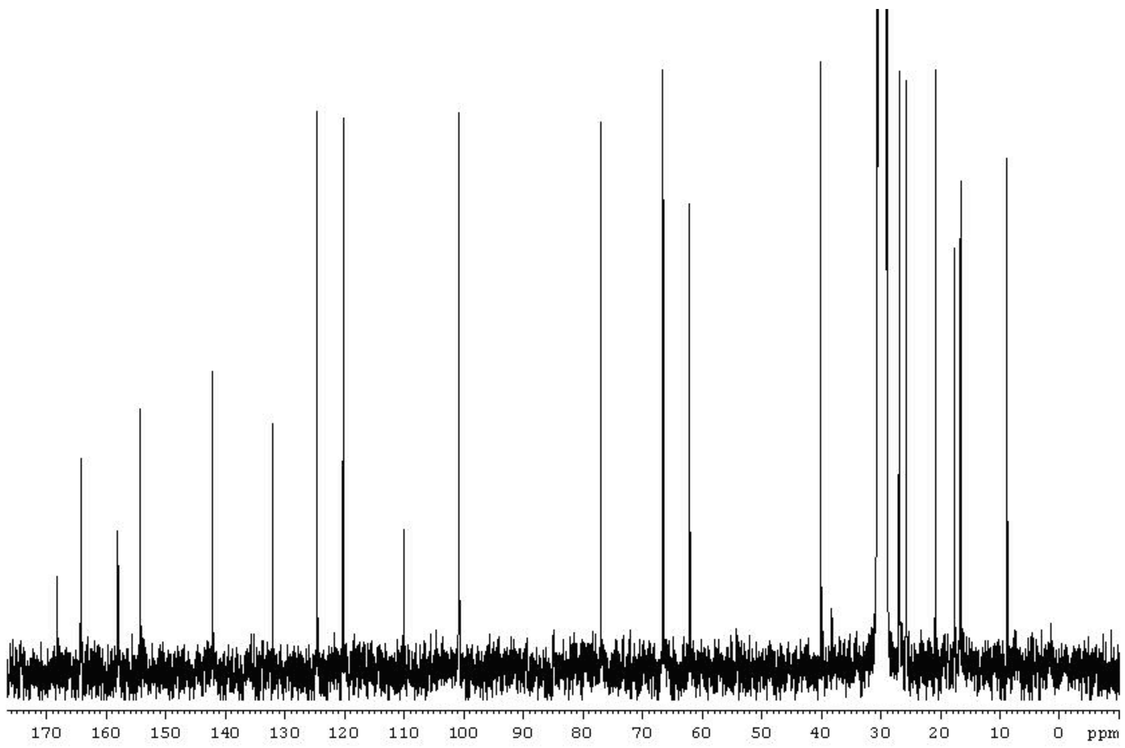

Figure S3:^{1}H NMR (300 MHz, CD_3COCD_3) of compound **2**.

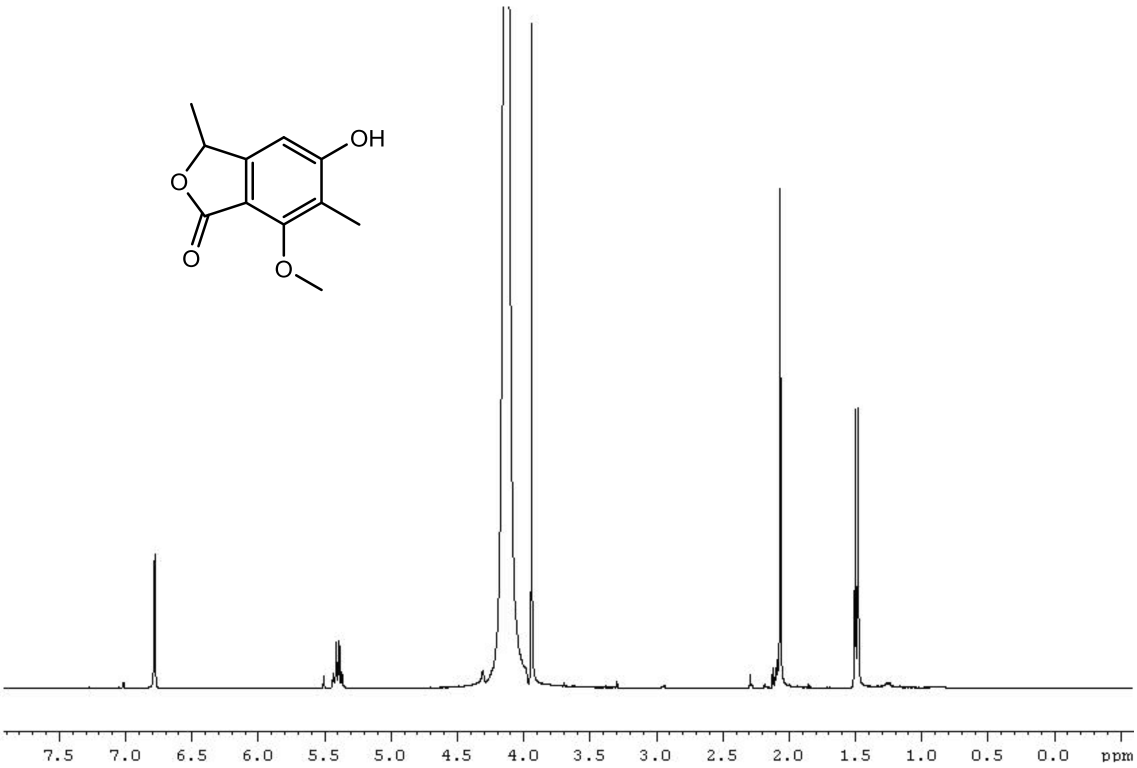

Figure S4:^{13}C NMR (75 MHz, CD_3COCD_3) of compound **2**.

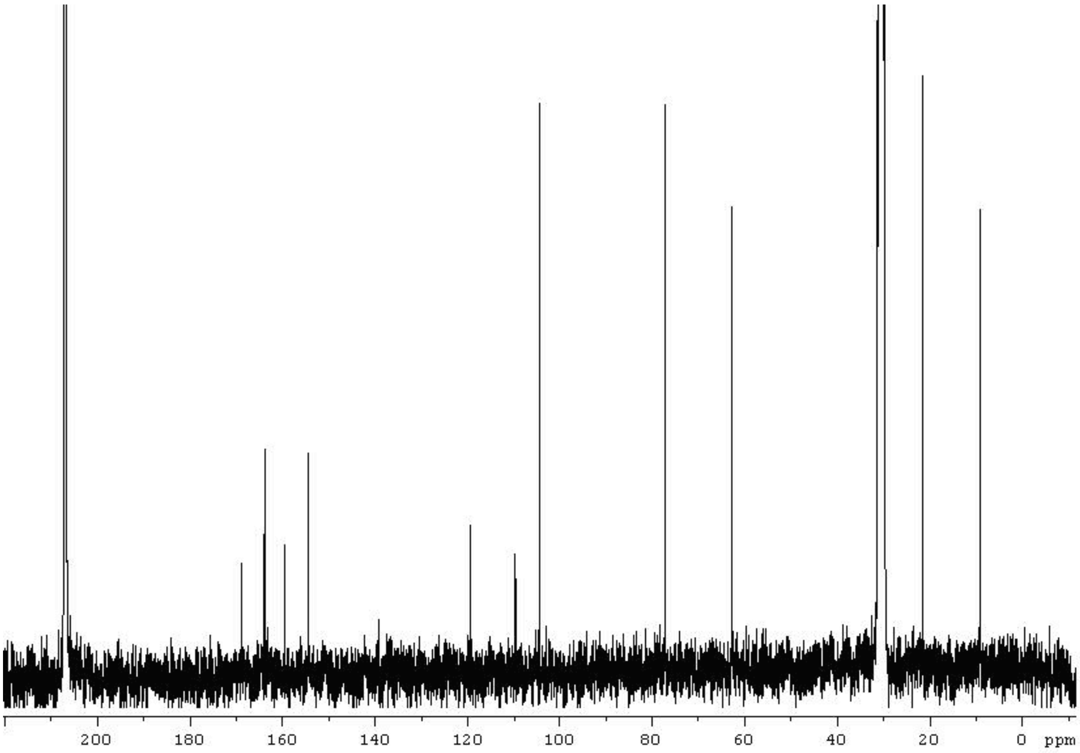

Figure S5: ^{1}H NMR (300 MHz, CD_3COCD_3) of compound **3**.

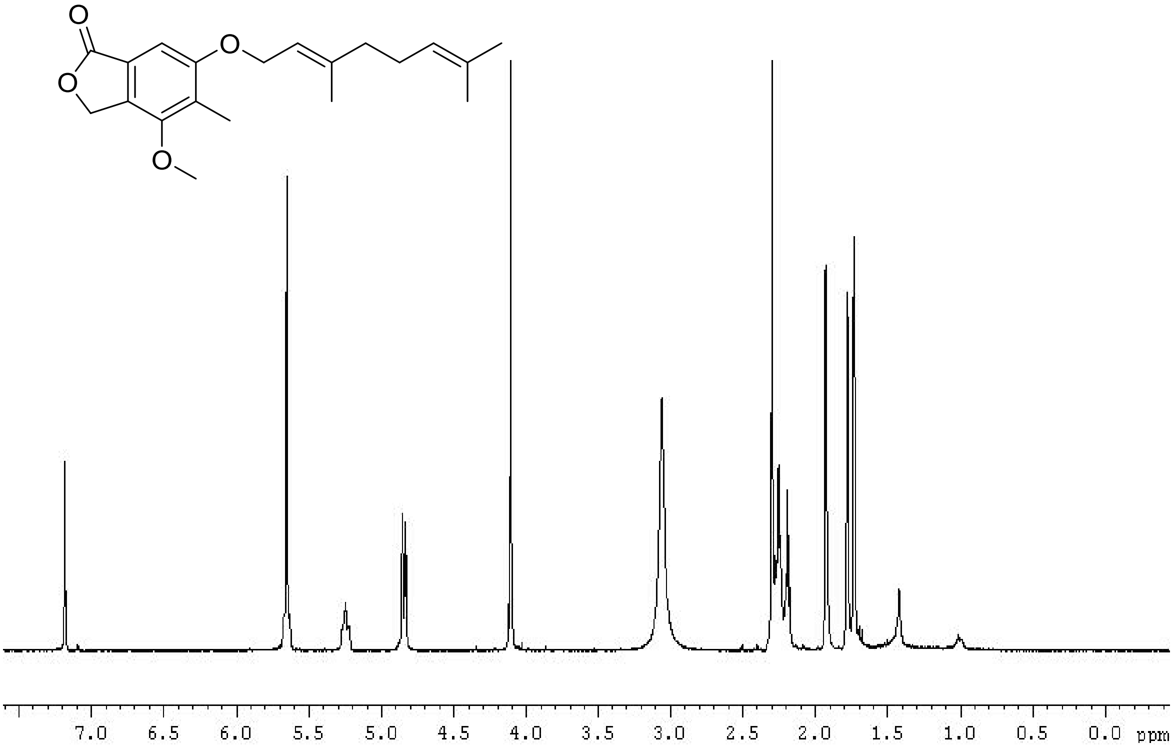

Figure S6: ^{13}C NMR (75 MHz, CD_3COCD_3) of compound **3**.

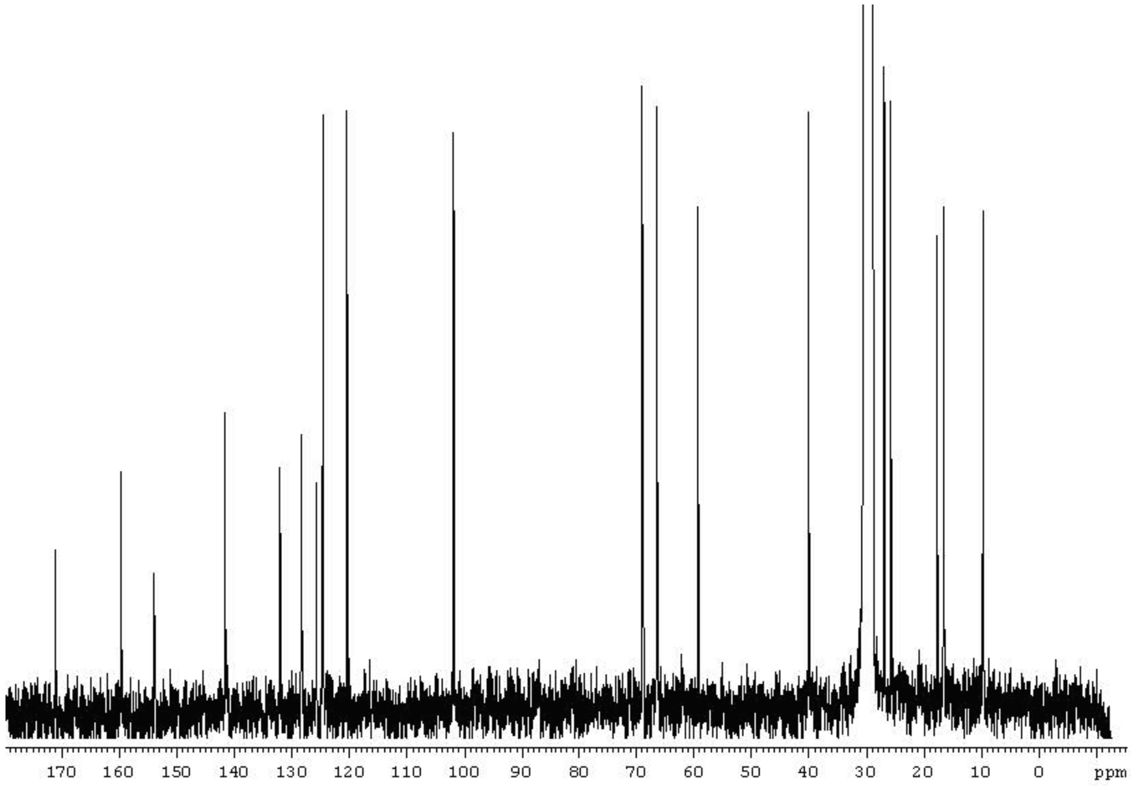

Figure S7: Key HMBC correlations for compounds **1**–**3**.

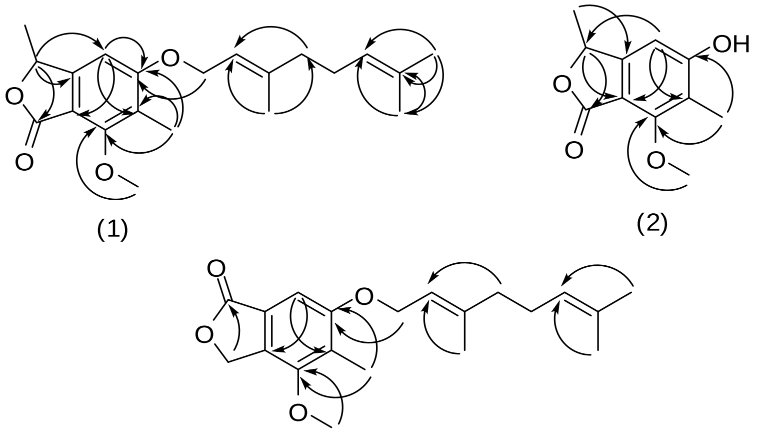

(1) (2)

(3)

Figure S8: HREIMS data for compound **1**.

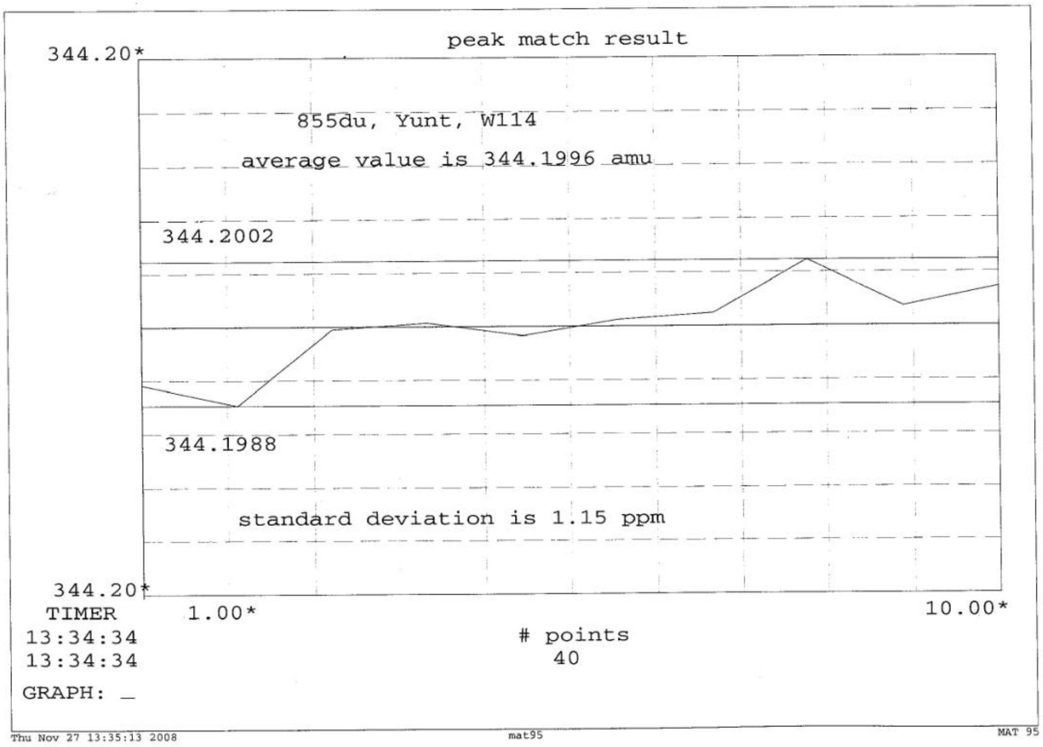

Figure S9: HREIMS data for compound **2**.

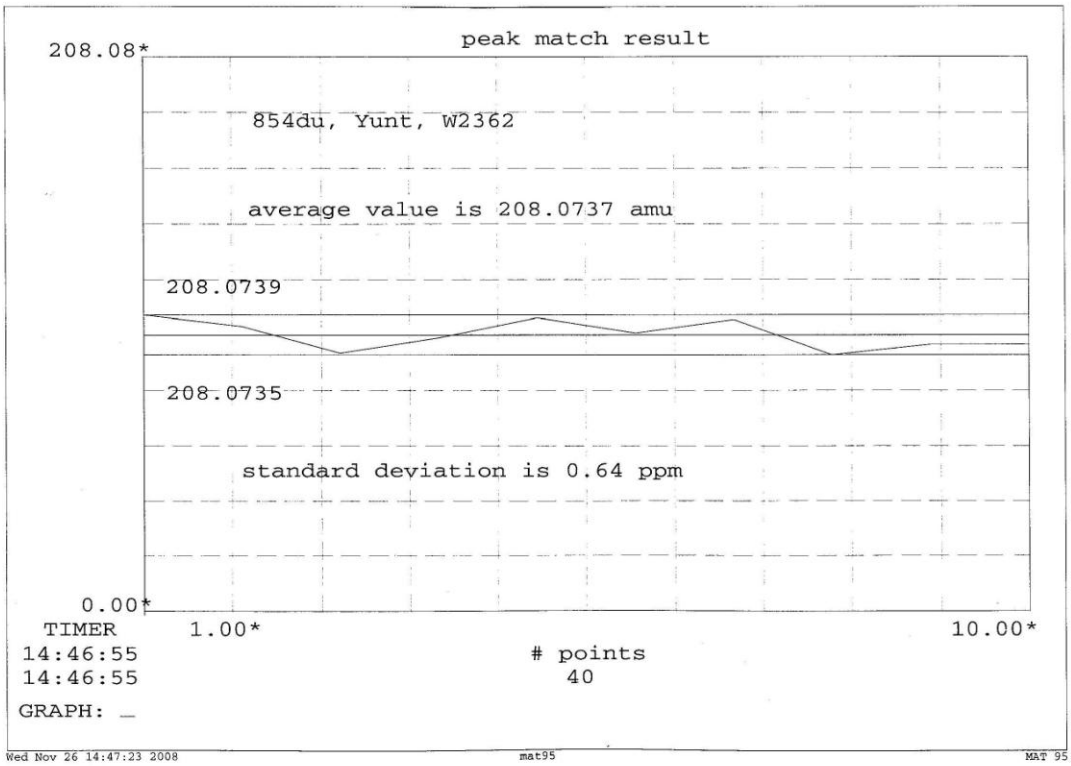

Figure S10: HREIMS data for compound **3**.

UCLR USER LIST FILE (4x500): 853du1 SAVE

1:	330.18349	0.00000	0.00000	0.00000
2:	330.18403	0.00000	0.00000	0.00000
3:	330.18132	0.00000	0.00000	0.00000
4:	330.18427	0.00000	0.00000	0.00000
5:	0.00000	0.00000	0.00000	0.00000
6:	0.00000	0.00000	0.00000	0.00000
7:	0.00000	0.00000	0.00000	0.00000
8:	0.00000	0.00000	0.00000	0.00000
9:	0.00000	0.00000	0.00000	0.00000
10:	0.00000	0.00000	0.00000	0.00000
11:	0.00000	0.00000	0.00000	0.00000
12:	0.00000	0.00000	0.00000	0.00000
13:	0.00000	0.00000	0.00000	0.00000
14:	0.00000	0.00000	0.00000	0.00000
15:	0.00000	0.00000	0.00000	0.00000
16:	0.00000	0.00000	0.00000	0.00000
17:	0.00000	0.00000	0.00000	0.00000
18:	0.00000	0.00000	0.00000	0.00000
19:	0.00000	0.00000	0.00000	0.00000
20:	0.00000	0.00000	0.00000	0.00000
LINK	NONE	NONE	NONE	NONE
SIZE	4.00000	0.00000	0.00000	0.00000
MEAN	330.18328	0.00000	0.00000	0.00000
SUM	1320.73312	0.00000	0.00000	0.00000
S.D.	0.00134	0.00000	0.00000	0.00000
MAX	330.18427	0.00000	0.00000	0.00000
MIN	330.18132	0.00000	0.00000	0.00000

ULIST: _

Wed Nov 26 15:10:58 2008 mat95 MAT 95

Figure S11: Antiproliferative activity data for compounds **1** and **3**.

Growth delaying activity (represented by GI_{50}), cytostatic activity (represented by TGI) and cytotoxic activity (represented by LC_{50}) were observed. GI_{50} is different from the IC_{50} in that it contains a correction for the starting population. Results in μM.

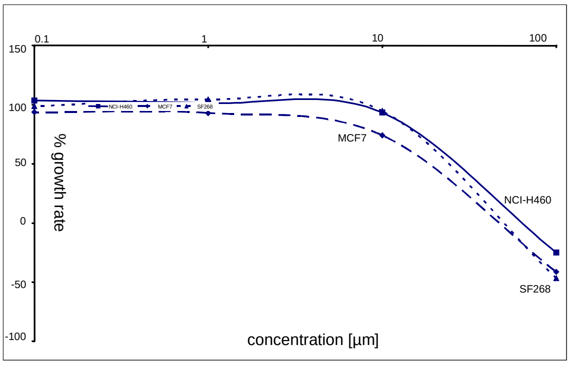

GI_{50} compound **1**

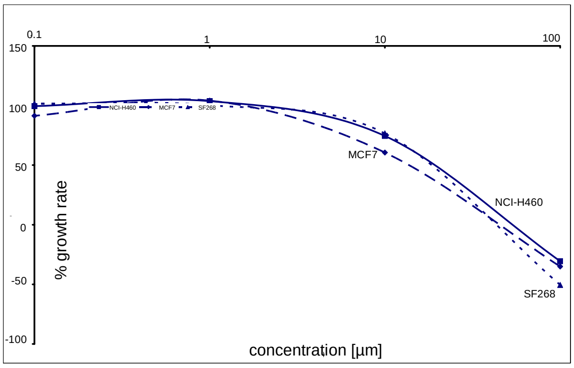

GI_{50} compound **3**

CELL LINES		**(1)**	**(3)**	**Etoposide**
**NCI-H460**	***GI_{50}***	42.9	31.0	0.4
	TGI	80.9	73.8	22.4
	LC_{50}	>100	>100	124.2
**MCF7**	***GI_{50}***	28.8	20.0	22.8
	TGI	67.7	67.0	118.0
	LC_{50}	>100	>100	213.1
**SF268**	***GI_{50}***	38.4	28.9	7.7
	TGI	70.2	64.2	90.4
	LC_{50}	>100	99.6	201.4

Cancer cell lines: NCI-H460/lung, MCF7/breast and SF268/CNS
Average values in main article.

Figure S12a–c: Antiplasmodial activity data for compounds **1** and **3**.

The bioactivity test monitors the inhibition of the infection of *Plasmodium berghei* liver stages. The parasite expresses luciferase, thus, the infection is measured by luminescence (Bars); the cellular density (i.e., the nontoxicity of the compounds) is measured by AlamarBlue fluorescence.

The tests were performed in triplicate measurements per compound concentration.

Samples were dissolved in acetone, which was also tested on its own. Results show that the solvent has no effect on cell confluency or the infection (the X axis of the acetone graphic depicts the amount of acetone present in the corresponding compound concentrations). The data show that whereas Marilone B does not affect infection of liver cells by *Plasmodium berghei*, Marilone A displays a dose-dependent effect on infection, with an IC50 of 12.1 μM, and without any cell toxicity at the concentrations used in the assay.

Figure S12a: Acetone measurements.

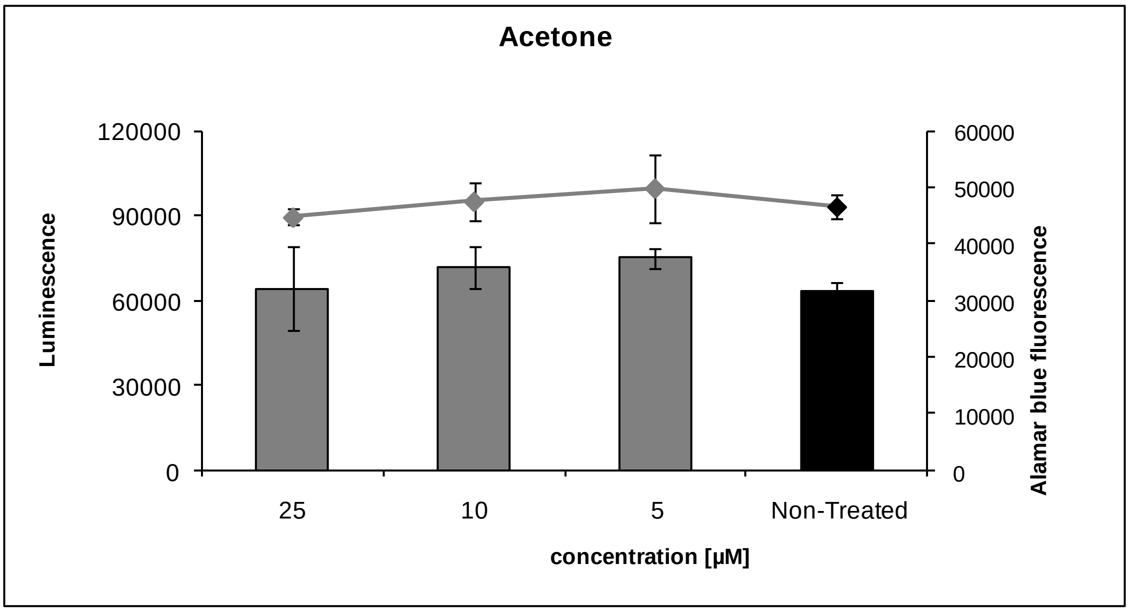

Figure S12b: Compound **1**.

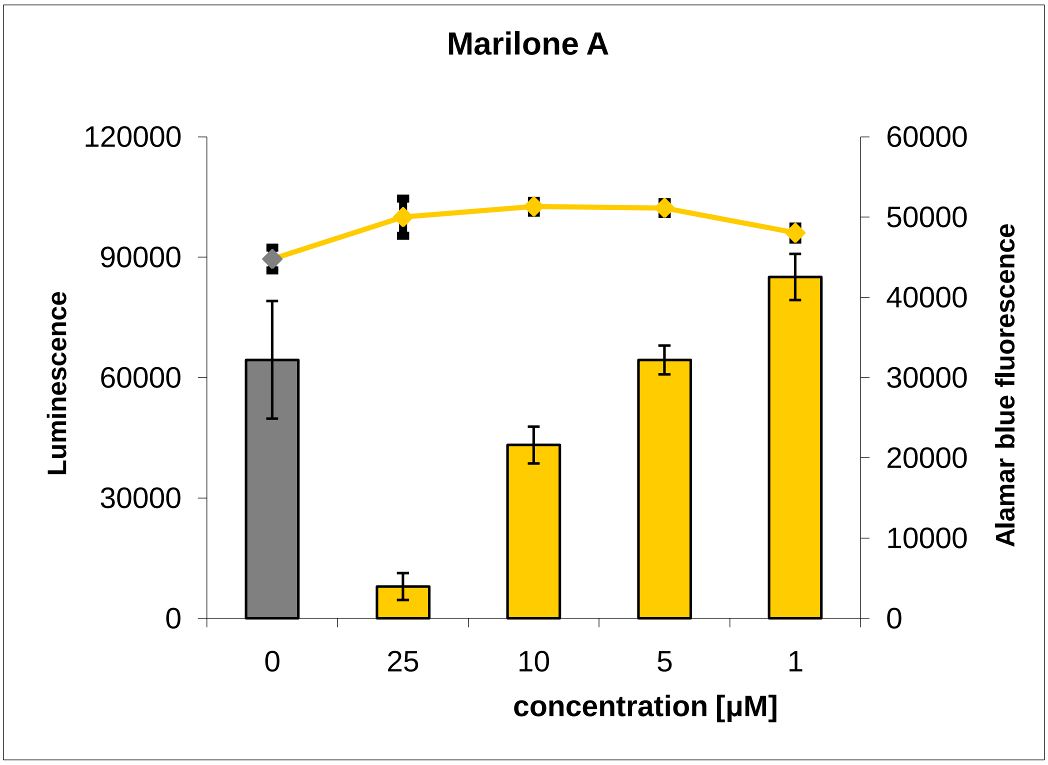

Figure S12c: Compound **3**.

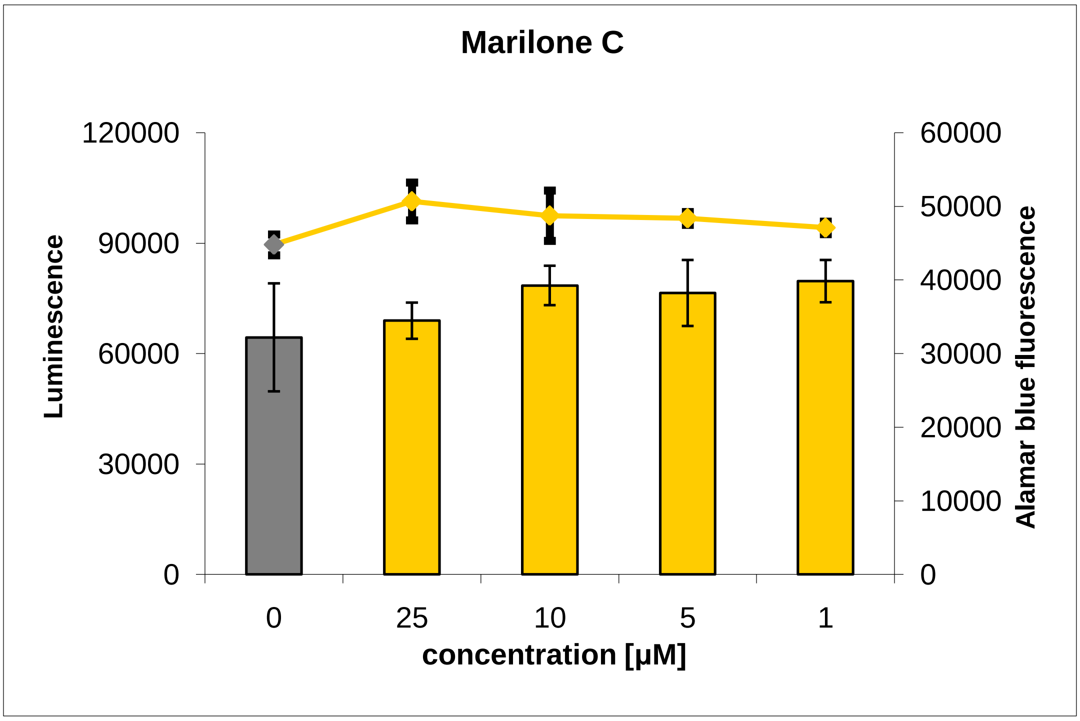

Figure S13: K_i value determination of the antagonistic effect of Marilone B (compound **2**) against the serotonin receptor 5HT2B.

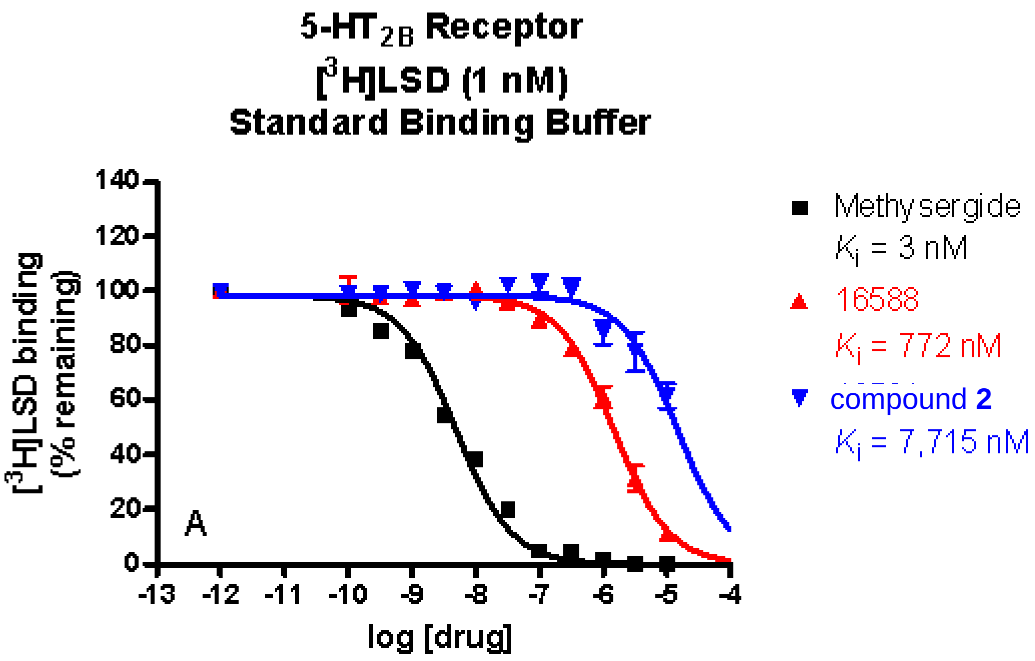

S14 Detailed description of the negative biological activity assays

Marilones A, B and C were tested in protein kinases (DYRK1A and CDK5) inhibition activity assay, inhibition of the viral HIV-1- and HIV-2-induced cytopathogenic effect in MT-4 cells, in antibacterial (*Escherichia coli*, *Bacillus megaterium*), antifungal (*Mycotypha microspora*, *Eurotium rubrum*, and *Microbotryum violaceum*), and antialgal (*Chlorella fusca*) assays at the 50 µg/mL disc level; they were evaluated for inhibition of the following panel of proteases: Chymotrypsin, trypsin, the protease elastase HLE, papain, porcine cease and acetylcholine esterase (tested at the 100 µM level) but exhibited no activity in any of these tests.

Marilones A and C were also evaluated against 3T3-L1 murine adipocytes assay and exhibited no activity.

Marilones A and B were evaluated against three subtypes of Influenza A virus (H1N1, H5N1 and H3N2), Influenza B virus (Flu B) and exhibited no activity.

Marilone A was evaluated against the Severe Acute Respiratory Syndrome coronavirus (SARS) and Hepatitis B virus but did not show any activity.

Marilone B was evaluated against the Herpes Simplex Virus-2 (HSV-2) and the Respiratory Syncytial virus (RSV) and exhibited no activity.

Marilone A was further tested against two antibiotic resistant *Mycobacterium tuberculosis* strains, MTB72 and R46 (tested at the 50 µg/mL disc level), but did not show any relevant activity.

Marilone C was tested in a panel of antidiabetic activity assays: PPAR ligand assay, PPAR co-activator assay, PTP-1B inhibitor assay and glucose uptake assay (both 3T3-l1 cells and primary rat/mouse adipocytes, tested at the 100 µM dose), but did not show any activity.

Marilone C was tested for the inhibition of the NF-kB protein complex assay (at the 100 µM), but did not show any activity.

Table S1: 1D and 2D NMR spectroscopic data for compound **1**.

position	δ_C, mult.[a,b,e]	δ_H[a,b] (*J* in Hz)	COSY[a,c]	HMBC[a,d]
1	168.2, qC			
2	110.0, qC			
3	158.0, qC			
4	120.4, qC			
5	164.2, qC			
6	100.8, CH	6.95, s		2, 3, 4, 5, 7, 8
7	154.2, qC			
8	77.0, CH	5.43, q (6.6)	11	1, 2, 6, 7, 11
9	62.1, CH_3	3.98, s		3
10	8.8, CH_3	2.09, s		3, 4, 5
11	20.9, CH_3	1.54, d (6.6)	8	7, 8
1'	66.5, CH_2	a: 4.69, dd (6.6, 12.1)	1’b, 2'	5, 2', 3'
		b: 4.74, dd (6.6, 12.1)	1’a, 2’	5, 2’, 3’
2'	120.1, CH	5.52, t (6.6)	1'a, 1’b, 9'	1', 4', 9'
3'	142.1, qC			
4'	40.1, CH_2	2.10, m	5'	2', 6'
5'	26.9, CH_2	2.13, m	4', 6'	3', 4', 6', 7'
6'	124.64, CH	5.09, m	4', 5', 8', 10'	4’, 5'; 8’
7'	132.1, qC			
8'	25.8, CH_3	1.63, br s	6'	6', 7'
9'	16.7, CH_3	1.76, br s	2’	2', 3', 4'
10'	17.7, CH_3	1.58, br s	6'	6', 7'

[a]CD_3COCD_3, 300/75.5 MHz. [b]Assignments are based on extensive 1D and 2D NMR experiments (HMBC, HSQC, COSY). [c]Numbers refer to proton resonances. [d]Numbers refer to carbon resonances. [e]Implied multiplicities determined by DEPT.

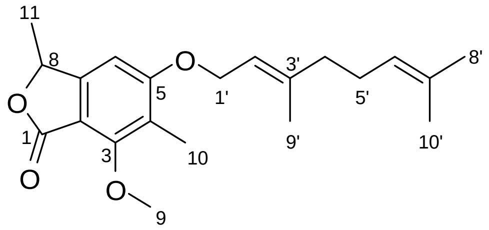

Table S2: ^{1}D and ^{2}D NMR spectroscopic data for compound **2**.

position	δ_C, mult.[a,b,e]	δ_H[a, b] (J in Hz)	COSY[a, c]	HMBC[a, d]
1	168.2, qC			
2	109.1, qC			
3	158.9, qC			
4	118.8, qC			
5	163.2, qC			
6	103.6, CH	6.73, s		2, 3, 4, 5, 8
7	153.9, qC			
8	76.6, CH	5.43, q (6.6)	11	1, 2, 6, 7, 11
9	62.0, CH_3	3.89, s		3
10	8.6, CH_3	2.02, s		3, 4, 5
11	21.0, CH_3	1.44, d (6.6)	8	7, 8

[a]MeOD, 300/75.5 MHz. [b]Assignments are based on extensive 1D and 2D NMR experiments (HMBC, HSQC, COSY). [c]Numbers refer to proton resonances. [d]Numbers refer to carbon resonances. [e]Implied multiplicities determined by DEPT.

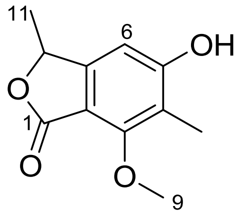

Table S3: ^{1}D and ^{2}D NMR spectroscopic data for compound **3**.

position	δ_C, mult.[a,b,e]	δ_H[a,b] (J in Hz)	COSY[a,c]	HMBC[a,d]
1	68.9, CH_2	5.50, s		2, 3, 5, 7, 8
2	128.4, qC			
3	153.9, qC			
4	124.8, qC			
5	159.7, qC			
6	102.0, CH	7.03, s		2, 3, 4, 5, 7, 8
7	125.8, qC			
8	171.1, qC			
9	59.3, CH_3	3.96, s		3
10	9.8, CH_3	2.15, s		3, 4, 5
1'	66.4, CH_2	4.71, d (6.6)	2'	5, 2', 3'
2'	120.5, CH	5.51, t (6.6)	1', 9'	4', 9'
3'	141.6, qC			
4'	40.1, CH_2	2.10, m		2', 3', 6'
5'	26.9, CH_2	2.14, m	4', 6'	3', 4', 6', 7'
6'	124.6, CH	5.10, m	4', 5', 8', 10'	4', 8', 10'
7'	132.05, qC			
8'	25.8, CH_3	1.63, br s	6'	6', 7', 10'
9'	16.7, CH_3	1.78, br s	2'	2', 3', 4'
10'	17.7, CH_3	1.58, br s	6'	6', 7', 8'

[a]CD_3COCD_3, 300/75.5 MHz. [b]Assignments are based on extensive 1D and 2D NMR experiments (HMBC, HSQC, COSY). [c]Numbers refer to proton resonances. [d]Numbers refer to carbon resonances. [e]Implied multiplicities determined by DEPT.

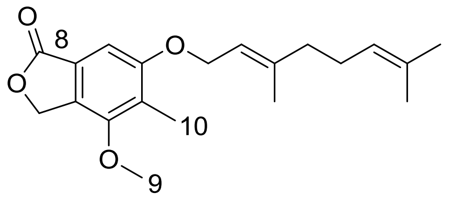

Table S4: ^{1}H NMR spectroscopic data comparison of compound **4** with silvaticol and nidulol.

	^{1}H NMR ($CDCl_3$)		
Position	Compound **4**	Silvaticol[1]	Nidulol[1]
1			
2			
3			
4			
5			
6	**7.05**	**7.04**	**6.59**
7			
8	5.39	5.39	5.15
9	3.90	3.91	4.08
10	2.23	2.23	2.19

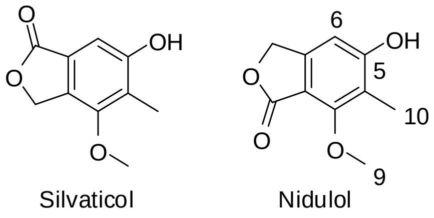

1. Kawahara, N.; Nozawa, K.; Nakajima, S.; Udagawa, S.; Kawai, K. *Chem. Pharm. Bull.* **1988**, *36*, 398-400.

Table S5: ^{1}H NMR spectroscopic data comparison of compounds **1** and **3** with silvaticol and nidulol derivatives.

			^{1}H NMR ($CDCl_3$)	
position	**1**	**3**	6-(3',3'-dimethylallyloxy)-4-methoxy-5-methylphthalide[2]	5-(3',3'-dimethylallyloxy)-7-methoxy-6-methylphthalide[2]
1				
2				
3				
4				
5				
6	**6.54**	**7.05**	**7.08**	**6.62**
7				
Protons at				
C-1/C8	5.39	5.39	5.38	5.18
9	4.04	3.90	3.89	4.03
10	2.18	2.23	2.21	2.15
			silvaticol derivative	nidulol derivative

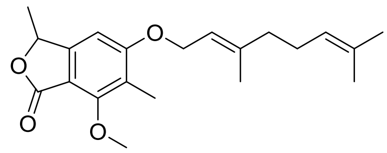

Compound 1

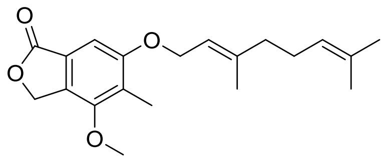

Compound 3

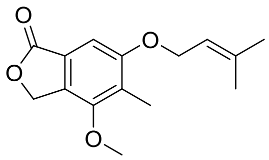

silvaticol derivative

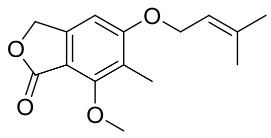

nidulol derivative

2. Suemitsu, R.; Ohnishi, K.; Morikawa, Y.; Nagamoto, S. *Phytochemistry* **1995**, *38*, 495-497.

Figure S15: Tetraketide nature of the phthalides nidulol (A) and silvaticol (B), and hypothetical biosynthetic pathways (C, D) for marilones A and B (**1**, **2**).

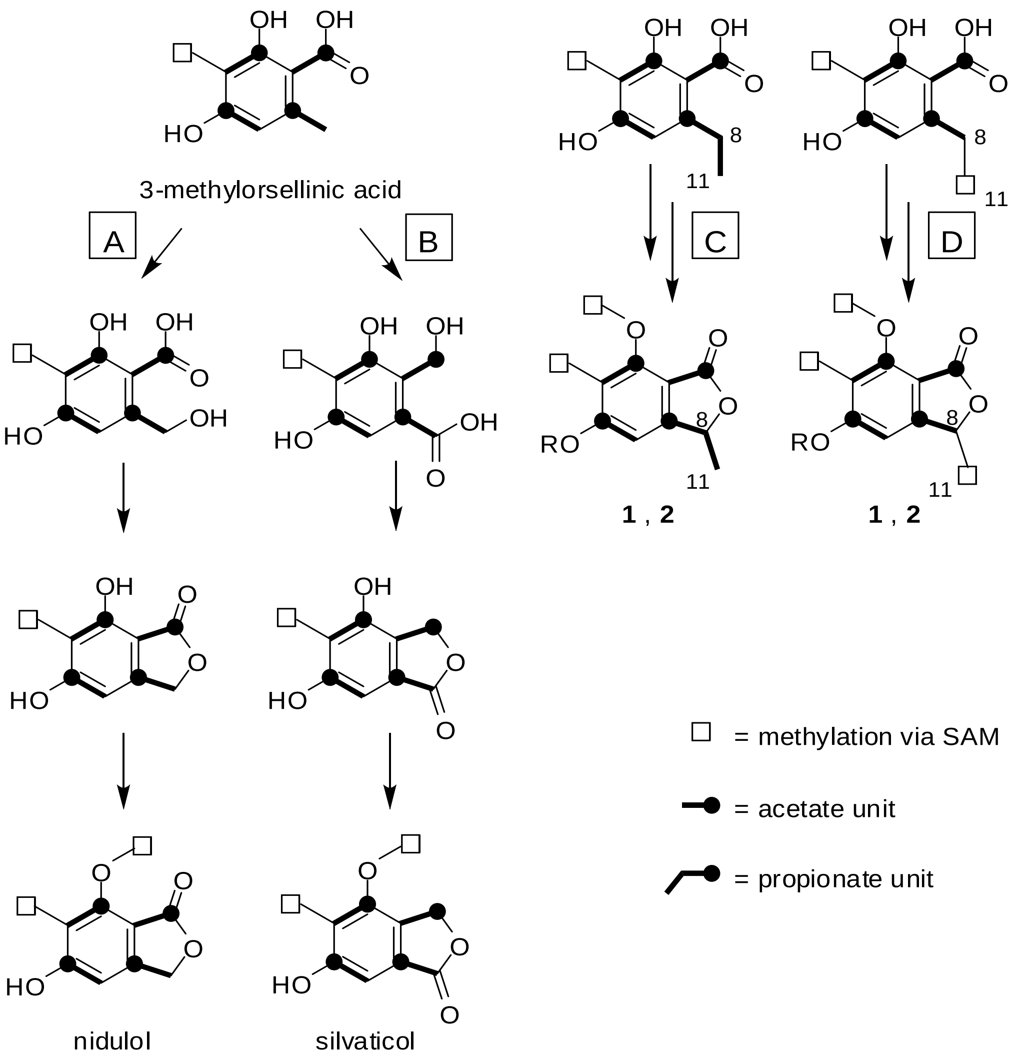

Supplement: File 1 — Spectroscopic data and other relevant information for compounds 1–4. [file Beilstein_J_Org_Chem-07-1636-s001.pdf]
